# Effect of a mindfulness training app on a cigarette quit attempt: an investigator-blinded, 58-county randomized controlled trial

**DOI:** 10.1093/jncics/pkad095

**Published:** 2023-11-10

**Authors:** David S Black, Matthew G Kirkpatrick

**Affiliations:** Department of Population and Public Health Sciences, Keck School of Medicine, University of Southern California, Los Angeles, CA, USA; Norris Comprehensive Cancer Center, Cancer Control Research Division, Keck Medicine of USC, Los Angeles, CA, USA; Department of Population and Public Health Sciences, Keck School of Medicine, University of Southern California, Los Angeles, CA, USA; Norris Comprehensive Cancer Center, Cancer Control Research Division, Keck Medicine of USC, Los Angeles, CA, USA; Department of Psychology, University of Southern California, Los Angeles, CA, USA

## Abstract

**Background:**

Cigarette smoking is the leading cause of preventable cancers. A majority of the 34 million people who currently smoke report wanting to quit. Mindfulness training apps offer a guided telehealth intervention to foster individuals’ behavioral meditation practice. We present the main outcomes of a parallel-group randomized controlled trial that tested app-based mindfulness training vs attention control on smoking behavior.

**Methods:**

We enrolled adult residents from across California who smoked daily and were willing to make a quit attempt (N = 213). Participants completed daily sessions in 10-minute segments for 14 consecutive days. Participants then started a quit attempt and reported daily smoking for 28 days following the quit date using the timeline follow-back measure.

**Results:**

Seven-day point-prevalence abstinence for each week during the 4-week quit period ranged from 21.8% to 27.7% for app-based mindfulness training and 17.9% to 19.6% for controls. The intention-to-treat sample revealed that app-based mindfulness training outperformed controls on the proportion of abstinence days during the quit period (odds ratio = 2.00, 95% confidence interval = 1.03 to 3.87, *P* = .041). Although the 7-day point prevalence abstinence for week 4 favored app-based mindfulness training, significance was not reached (odds ratio = 1.65, 95% confidence interval = 0.84 to 3.23, *P* = .148). The mean number of cigarettes smoked per day among smokers was 4.95 for app-based mindfulness training vs 5.69 for controls (odds ratio = 0.81, 95% confidence interval = 0.71 to 0.92, *P* = .002), suggesting harm reduction in continued smokers.

**Conclusion:**

A mindfulness training app prescribed for 2 weeks leading up to a quit date showed an advantage over controls for total abstinence days and fewer cigarettes smoked in a diverse sample consisting of urban and rural residents. These findings yield implications for the use of apps to reduce exposure to the carcinogenic properties of cigarette smoke.

##  

Cigarette smoking is connected to a multitude of diseases and is the foremost contributor to preventable cancers. It exacts a toll of nearly 500 000 lives each year and imposes an annual health-care expenditure in excess of $300 billion (1,2). Despite these alarming statistics alongside ubiquitous public health messaging about the harms of smoking, there are still an estimated 34 million cigarette smokers in the United States, with the majority expressing a desire to quit smoking for health reasons (3). This high rate of failure in quit attempts can be attributed to the fact that many of these attempts are self-initiated and unassisted, lacking the support of a program, medication, or counselor (4). Given this reality—where most quit attempts are unassisted and result in relapse (5)—it is imperative to develop low-cost, easily accessible behavior interventions to support individuals striving for complete cessation and harm reduction. Smartphone apps are a promising telehealth option to address this issue. To reach a broad range of smokers in both rural and urban areas with varying levels of access to support programs and resources, we propose that current smokers who wish to quit may benefit most from easily accessible remote behavior interventions delivered through a smartphone app.

Mindfulness training encompasses intervention packages that provide individuals with meditation training and encourage them to incorporate mindfulness into their daily lives. The instructional content of mindfulness training guides individuals to simply observe sensory and mental states (eg, discomfort, itchiness, pain, urges, thinking, worrying) as they arise and fade away during meditation sessions, all without making associated responses (eg, moving, scratching, thinking about thinking, mental problem solving) (6). As individuals engage in the practice of experiencing fluctuating sensory and mental states in their meditation sessions without generating corresponding responses, any response previously associated with that state is weakened (eg, itchiness is less likely to elicit scratching; a smoking urge is less likely to elicit smoking) (7). This general behaviorist principle has previously been explained in terms of “urge surfing” in the arena of smoking cessation (8). In terms of observable behavior, meditators are instructed to adopt a comfortable posture during meditation, such as lying down, sitting, standing, slow walking, yoga, or engaging in light stretching (6).

To apply the principle of nonresponse during mindfulness training sessions—a process of weakening behavior to enhance smoking cessation success in the population of smokers willing to make a quit attempt—we proposed introducing app-based mindfulness training to individuals before their voluntary quit attempt. This pre-quit period offers time to practice mindfulness training and cultivate nonresponse during meditation to weaken the probability of a smoking response when in a state of smoking abstinence and associated nicotine/tobacco deprivation [see Skinner (9)]. The aversive states that smokers experience when making a quit attempt commonly include cravings to smoke, restlessness, increased appetite, anxiety, constipation, negative affect, and difficulty concentrating. Mindfulness training is 1 approach that allows individuals making a quit attempt to experience abstinence and its associated discomforts without responding by smoking; thus, mindfulness training potentially supports a greater likelihood of complete abstinence, more abstinence days, and fewer cigarettes smoked.

The research literature, in general, supports a net benefit to human health associated with participation in various mindfulness training programs, such as mindfulness-based stress reduction and mindfulness-based cognitive therapy, in both normative and clinical samples (10,11). Empirical tests of mindfulness-based interventions are growing in the field of treatment for substance use disorders (12-14), but relatively less is known about the effect of mindfulness training on smoking cessation among people who smoke and are willing to make a voluntary quit attempt while being assisted by a smartphone app. A meta-analysis of randomized controlled trials suggested that mindfulness training intervention assignment is associated with an increased likelihood of remaining abstinent after a smoking quit attempt (15), yet a more recent review from the Cochrane Library concluded with low confidence that the effect of mindfulness training is sufficiently robust for smoking cessation (16). Experimental studies show that abstinent individuals display less craving response to an anxiety-provoking task, a common trigger for smoking lapse, after a single 7-minute mindfulness meditation bout (17,18).

In an efficacy trial that compared treatment packages, adults with nicotine dependence were randomly assigned to receive 4 weeks of in-person, group-based mindfulness training adapted specifically for smoking cessation or the American Lung Association’s cognitive behavior therapy program (19). The mindfulness training group demonstrated significantly greater 7-day point-prevalence abstinence at 4-month follow-up (31% vs 6%). In a separate randomized controlled trial comparing remote delivery of mindfulness training using a mobile app—again, specifically adapted for smoking cessation—with mobile app monitoring only (ie, experiential sampling of smoking behavior and related symptoms), both study groups showed a significant reduction in the number of cigarettes smoked per day; however, there was no significant difference in proportion between groups in terms of 7-day point-prevalence abstinence at 6-month follow-up (mindfulness training group = 10% vs control = 12%) (20). When we consider the evidence in the field to date, the available literature indicates a positive effect for mindfulness training to support harm reduction, defined as fewer cigarettes smoked, but mixed effects for 7-day point-prevalence abstinence. It is unknown whether generalized mindfulness training apps not adapted for smoking have a similar effect on smoking outcomes and in a more diverse sample. The rationale for conducting a test of generalized mindfulness training is based on the absence of studies comparing this type of program with a standard care control group among smokers. Consequently, it remains uncertain whether additional smoker-specific adaptations are necessary beyond the typical language associated with mindfulness practices applied in daily life on a broader scale. This reasoning holds significance for smokers who are keen to quit but would rather not highlight smoking as a central part of their journey toward a healthier lifestyle.

### Study objective

Our study objective was to test the effect of a commercially established mobile app–based mindfulness training telehealth intervention focused on guided mindfulness meditation but not specific to smoking cessation relative to a time-matched mobile attention control on smoking among people who currently smoke and are willing to make a quit attempt. In this remotely administered study, we recruited people who were current smokers from all 58 counties in California. Our daily delivery of the interventions for 2 weeks leading up to a planned quit date provided individuals who smoke the opportunity to prepare for their quit date. We used an investigator-blinded, parallel, between-group, randomized controlled trial design to test the hypothesis that the app-based mindfulness training group would show a greater proportion of smoking abstinence (complete 7-day point-prevalence abstinence and total days abstinent) and show fewer cigarettes smoked per day among those who continue to smoke (secondary harm reduction outcome) during the 28 days following the quit attempt start date. We anticipated that our findings could uniquely contribute to the fields of medicine and public health by determining whether a low-demand mindfulness training stand-alone app not specific to smoking behavior or craving symptoms—as proposed in previous studies (20)—could improve smoking cessation and harm reduction and so reduce exposure to the carcinogenic properties of cigarette smoke (21).

## Methods

### Study design

This parallel-group, outcomes assessor–blind, randomized controlled trial involved participants recruited from both urban and rural regions across all 58 counties of California. The trial aimed to assess the efficacy of daily, app-based behavior interventions in helping people who smoke daily quit or reduce smoking. The pretrial protocol was published for review (22). Two intervention groups were included: an app-based mindfulness training package and an attention control package, with the control accounting for extraneous factors elicited from research protocol exposure, such as the participant’s time, attention, and expected benefit. Participants were asked to self-administer their sessions twice a day in 10-minute segments for 14 consecutive days, for a total of 280 minutes (ie, the adherence denominator). The trial was conducted between July 2021 and December 2022, amidst the COVID-19 global pandemic, and was registered with ClinicalTrials.gov (NCT05440903) and institutional review board approved (UP-20-00900). Smoking behavior was assessed, with the timeline follow-back calendar completed by computer at day 28 after the quit date (see “Measures”), which is the day when the participants completed their participation in the trial.

### Participants and procedures

Individual adults were recruited online through Craigslist advertisements, and participants completed all study protocols and interviews remotely from their preferred location using a secure online videoconferencing platform. This method extended our reach of recruitment and addressed the in-person limitations caused by the COVID-19 pandemic and associated state and local social isolation policies that restricted in-person interactions. Individuals who viewed the study advertisement followed a link to complete an online survey to demonstrate interest in the study, after which they made a voluntary request to be contacted by the study team. The study staff contacted those individuals with positive screens by phone to interview each person and confirm their eligibility for the study. A 15-minute phone screening was used to determine initial study eligibility and to schedule a baseline interview for those who passed the screening phase. During the screening call, the staff confirmed the participant’s current smoking status (eg, smoking 5 or more cigarettes per day for the past 2 years). Those who passed screening received electronic informed consent documents to e-sign and return after completing a phone-based verbal informed consent process, which was led by a trained member of the study staff.

After providing consent, individuals were assessed for additional eligibility criteria during a baseline videoconferencing interview led by trained study staff. Those who met the criteria transitioned to participant status and received the baseline survey to complete before their remote interview with the study staff. The baseline interview lasted approximately 120 minutes, during which participants completed surveys, participated in a 20-minute motivational interview counseling session, and received instruction on study participation and when to start their quit attempt. This motivational interviewing session was conducted by trained staff members, who provided all participants with a 20-minute smoking cessation counseling session. The session was structured into 5 modules covering the following areas: 1) tobacco use and past quit attempts, 2) level of dependence and withdrawal symptoms, 3) smoking triggers and strengths and barriers regarding quitting, 4) quit strategies, and 5) anticipating and planning for lapses. Participants also received an electronic copy of the National Cancer Institute’s workbook, *Clearing the Air,* which contains additional information for preparing for a cessation attempt (23). This smoking cessation counselling ensured that all participants had a similar baseline knowledge of self-directed smoking cessation strategies and used evidence-based recommendations for tobacco dependence treatment.

At the end of the baseline interview, participants were enrolled in the trial and randomly assigned to a study group. Participants were informed that they could use their assigned app at will throughout the follow-up period. Study staff helped participants install and register the assigned app on their personal smartphone. Staff members who were unblinded to a participant’s study group did not complete the outcome assessment for that participant.

Participants voluntarily completed their daily app-based intervention for 14 days, and then attended a postintervention interview on their scheduled quit date. The immediate postintervention interview included verbal initiation of the voluntary quit attempt as well as completion of surveys and interviews. All app content remained available for use by participants during the postintervention follow-up period. Participant compensation reached a possible maximum of $300: completing the screening baseline intake survey ($30), baseline interview ($40), daily phone prompt reporting of the number of cigarettes smoked ($15 possible per week across 28 days, for a maximum of $60), daily audio app training ($2.50 for each of 28 possible app-based intervention sessions over 14 days, for a maximum of $70 paid as a lump sum at the postintervention videoconferencing interview), postintervention videoconferencing interview ($40), and 28-day follow-up surveys ($20), with a $40 bonus for completing more than 80% of surveys across 28 days of daily diary assessments.

### Eligibility requirements

To be eligible for the study, individuals had to be 18 years of age or older, have smoked daily (at least 5 cigarettes per day) for the past 2 years, be willing to make a self-directed and voluntary cigarette smoking quit attempt during the study period, and be a current resident of California (verified by a state-issued ID, with a California mailing address). People were ineligible if they were not fluent in English, lacked access to remote video capability (such as a computer, camera, and internet or a smartphone), had an ongoing mindfulness or meditation practice of more than 5 minutes per day within the past 30 days, or had used any nicotine-replacement products (such as nicotine patch, gum, or lozenge) or smoking cessation medications (such as varenicline or bupropion) within 30 days of baseline. The exclusion of ongoing mindfulness practice of more than 5 minutes per day was necessary to test the effect of taking on a new study treatment (meditation) as the independent variable. Cessation aids were also excluded from the study to detect singular behavior intervention effects rather than interactions with pharmacologic effects.

### Randomization and blinding

Author M.K. used a computer-generated random selection of block permutations, with a range of 4 to 20 assignments per block, including strata to have sex equivalent, which allowed random assignment of 1 study intervention to each participant. This block method reduced potential selection bias, as with simple randomization, yet it offered the advantage to increase the likelihood of balanced allocation to groups (24). The study staff responsible for supplying the app to participants during the baseline interview were given the randomization list only with the assigned app. To eliminate staff expectations, staff members who trained a participant on app use were not outcomes assessors for that participant (25). Participants were unaware of their app assignment until the end of the baseline interview, when they were instructed to use the assigned app. The investigators were blinded to trial datasets that included the group assignment variable during the active trial and up to the completion of statistical analysis for the trial outcomes. To eliminate investigator expectations as an extraneous factor, a third-party statistician, guided by a priori decision rules, independently conducted the statistical tests that produced the trial results.

### Interventions

#### App-based mindfulness training

*App-based mindfulness training* refers to the commercially available Headspace software application recordings [https://www.headspace.com; the Foundation Pack; see the published trial protocol for details (22)], which provided prerecorded introductory mindfulness meditation audio instructions guided by experienced meditation teachers. The app included both didactic information about mindfulness meditation as well as supplied guided mindfulness meditation practice during each session. We did not make any study alterations to the commercially available sessions. Study participants were instructed to complete 10 minutes of Headspace twice per day for 14 days, which is a total recommended dose of 280 minutes. Headspace practice started on the day after the baseline interview (ie, intervention day 1). Participants were instructed to listen to the recordings (including didactics and guided mindfulness meditation) at 2 separate times each day to maximize daily exposure with low time burden. Study staff tracked adherence to the training regimen by positioning it as the number of intervention sessions an individual used out of a total of 28 possible sessions (2 sessions per day for 14 days), calculated as the number of total minutes divided by 28. It is important to note that Headspace, by design, tallies only the number of minutes each participant spent in mindfulness practice during each 10-minute session. Thus, all participants in the trial were assigned 10 minutes of app content per session, but in the mindfulness training group, the measured meditation time was systematically reduced as an artifact of data being recorded for meditation practice time only. As participants had little or no prior experience with mindfulness, all recommended training was at the beginner level. Participants had access to all sessions the app offered and were not restricted to a particular sequence of sessions during the 14 days or during the quit attempt period. Additionally, all participants in this group received a conventional smoking cessation workbook to complete (the National Cancer Institute’s *Clearing the Air*). This publication provides cessation information and worksheets to support a quit attempt (https://www.cancer.gov/publications/patient-education/clearing-the-air-pdf).

#### Attention control (app-control)

*App-control* refers to freely available audio recordings of TED Talks that provide psychoeducation on field-specific topics by select experts [https://www.ted.com; see the published protocol for details (22)]. Study participants were instructed to listen to 10 minutes of TED Talks twice per day for 14 days, with a total recommended dose of 280 minutes. The 28 preselected sessions delivered by hyperlink to participants were selected by the study team to be of interest to the general public while not including content on meditation, smoking, or other content that might cue behavior change associated with smoking. Like app-based mindfulness training, app-control sessions started on the day following the baseline interview (ie, intervention day 1). Participants were instructed to listen at 2 separate times each day to maximize daily exposure with low time burden. Additionally, participants were instructed to listen with “full mindful attention and return attention to the audio when attention drifts,” which matches the app-based mindfulness training instruction and emphasizes the importance of sustaining attention for each 10-minutes period. The instructional statement was planned to ensure that all participants paid close attention to the assigned audio content. It was intentionally designed to promote concentration on the material, although it does not constitute a genuine mindfulness strategy. Previous research shows that sham meditation language does not produce the same effects on the brain and behavior as mindfulness training does (26). As with the app-based mindfulness training condition, study staff tracked adherence to sessions. Participants had access to all sessions offered and were not restricted to a particular sequence of sessions during the 14 days or during the quit attempt period. As with the app-based mindfulness training group, all participants in the app-control group received a conventional smoking cessation workbook to complete—the National Cancer Institute’s *Clearing the Air* publication, which contains cessation information and worksheets to support a quit attempt.

### Measures

#### Cigarette smoking

The primary trial outcome was cigarette smoking abstinence, which was the target behavior captured in the timeline follow-back calendar, collected through Research Electronic Data Capture, and completed on day 28 following the quit date [which differs by 2 days due to a shortened measure to capture increments of 1 week for 7-day point-prevalence abstinence scoring from our original plan for a 30-day follow-up, as stated on ClinicalTrials.gov (NCT05440903) (27)]. There was considerable missingness of data from the phone-based prompting method for daily cigarettes, making daily analysis based on the daily phone surveys untenable. Therefore, by using the timeline follow-back calendar, we used smoking cessation at the individual level the 7-day point-prevalence abstinence, as defined previously (ie, not smoking a single cigarette in the previous 7 day period) (28), calculated for all weeks and for the final week of follow-up. We also quantified total days of abstinence during the overall 28-day follow-up period given the brief intervention that might function to elicit harm reduction rather than cessation. Finally, we quantified daily cigarette count from each participant, and we calculated mean daily cigarette count for each participant and analyzed this finding as a secondary outcome again to test for harm reduction among those who continued to smoke during the quit period (23).

#### Sample characteristics

We collected descriptive information about each participant on the study baseline survey to understand the characteristics of the sample obtained and to verify that randomization assigned characteristics to study groups in a similar manner. Baseline measures included demographic information, smoking history, the Fagerström Test for Cigarette Dependence (FTCD) (29), and the Five-Facet Mindfulness Questionnaire (30).

### Statistical analysis

#### Sample size calculation and data analysis

The primary trial outcome in the intent-to-treat (ITT) sample analysis was the between-group proportional difference in smoking abstinence (7-day point-prevalence abstinence and proportion of days abstinent). To guide our original decision on the size of the sample for the trial, we conducted an a priori power analysis using G*Power software (Heinrich-Heine-Universität Düsseldorf, Germany) (31). Based on previous trial findings using this same outcome and comparing mindfulness training with smoking cessation education in adults who smoke (32), we estimated the need for a total sample size of N = 200 participants (100 per study group) to detect a group aggregate–level proportional difference of medium size (Cohen *d* = 0.55), with a calibration of 80% power and a 2-tailed test with an ɑ cutoff at 5%. We oversampled at baseline to compensate for an anticipated overall 10% attrition rate.

Participant data were input and stored in the secured Research Electronic Data Capture system. Upon the trial's completion, the data were shared with a third-party statistician consultant from the same university. The purpose was to blind key study personnel and the investigators to the knowledge of group assignment during all analyses until the primary outcome tests were completed by an impartial statistician. Group contrast effects on the primary and secondary outcomes of the trial were carried out using the ITT analytic approach (33). We applied 2 methods for ITT analysis. The ITT-randomized analytic sample included all 213 participants randomized in the trial, and the ITT-single dose analytic sample included the 174 participants who completed at minimum a single app dose during the study intervention phase. Both ITT sample types offered conservative testing of trial effects relative to analysis of the per-protocol sample, which did not deviate impactfully from the original study protocol (34). Rather than suffering bias resulting from coding missing data points in the timeline follow-back calendar as smoking (35), missing data points were addressed by employing the full information maximum likelihood estimation procedure, which incorporates all available data from each participant without omitting cases from the analytic sample (36). As such, fewer assumptions were made about reasons for missingness. The results are described in terms of odds ratios (ORs) and number needed to treat derived from multivariate generalized estimating equation logistic models and incidence rate ratios derived from a generalized estimating equation Poisson model with associated 95% confidence intervals (CIs) and *P* values. Further, a Kaplan-Meier curve was generated with a statistical contrast test estimate to assess the number of abstinence days before return to first cigarette smoked. A significance level of .05 was used in relation to a 2-sided test for all analyses, and computations were made in Stata/SE, version 17.0 (StataCorp, College Station, TX). Data used in this analysis are freely available in Supplementary Material (available online) for this article, and all analysis codes are available from the first author (D.B.).

## Results

### Participant flow-through trial and sample characteristics

**Figure 1.**
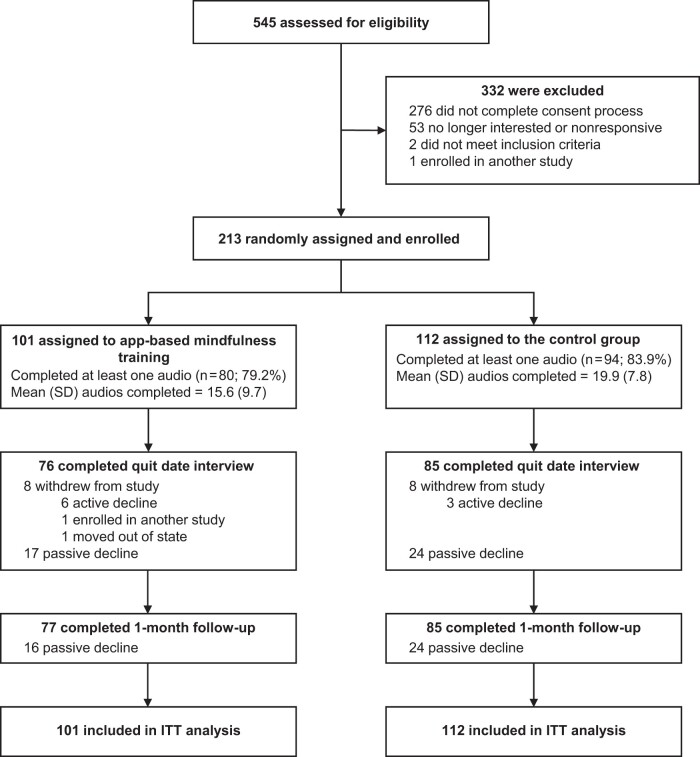
CONSORT diagram showing the flow of participants in the trial comparing app-based mindfulness training with controls. A total of 549 individuals completed an online study screener form and an e-signature consent form to be contacted by the study staff. Of those assessed by initial survey, 276 people did not finish the e-consent process. The phone-based apps allowed for automated recording of each participant’s app use, and records were visually inspected by study staff, who transcribed records to the dataset. The Headspace app interface provided the number of minutes each participant completed in open sessions and so did not include in the total minutes each session’s didactic preamble of instructions, which were variable in length, resulting in method artifact lower adherence values for app-based mindfulness training participants than for controls. ITT = intent to treat.

Figure 1 shows a CONSORT diagram depicting the flow of participants in the trial comparing the app-based mindfulness training group with controls. Of the 545 residents of California who passed the initial online screener for our study, 332 were excluded for ineligibility reasons, nonresponse, or loss of interest, and 213 were randomly assigned to a study condition and enrolled in the trial. In total, 161 completed their quit date interview assessment, conducted by Zoom, and 162 participants completed the 1-month follow-up assessment online from their self-selected environment. Six of 101 (5.9%) participants in the app-based mindfulness training actively declined, and 3 of 112 (2.7%) active controls actively declined and withdrew themselves from further study but allowed us to use their data for analysis. Those who passively declined were defined as those participants who ignored multiple contacts from the study team. The final outcomes assessment completion rate was 76% in each study group.

Table 1 provides descriptive variable values for the total sample and by group. There was no statistically significant group difference in mean scores for any measured variable shown at baseline. The mean age of those in the sample population was 41.2 years, with the majority being White (58%) and having an income less than $50 000 per year and one-quarter (25.4%) having a high school education or less. Before the intervention, the average (SD) number of cigarettes smoked per day in the total sample was 12.3 (6.1), and the mean (SD) FTCD score was 4.7 (2.0), a value indicating moderate dependence on average.

**Table 1.** Baseline descriptive variables for the randomized sample and by study group^a^

Variables at baseline	Total (N = 213)	App-based mindfulness training (n = 101)	Control (n = 112)
Age, mean (SD), y	41.2 (13.5)	41.9 (12.8)	40.6 (14.1)
Female sex at birth, %	53.9	55.5	54.5
Education, %			
Less than high school	3.3	4.0	2.7
High school diploma	22.1	21.8	22.3
Some college	48.4	48.5	48.2
College degree or higher	26.3	25.7	26.8
Income, %			
<$15 000	26.7	30.7	23.2
≥$15 000 to $29 999	28.2	25.7	30.4
≥$30 000 to $44 999	14.1	15.8	12.5
≥$45 000 to $74 999	18.3	17.8	18.8
≥$75 000	12.7	9.9	15.2
Race or ethnicity, %			
American Indian and/or Alaskan Native	1.9	1.0	2.7
Asian	2.4	1.0	3.6
Black	6.6	5.0	8.0
White	57.8	58.4	57.1
Other	3.3	4.0	2.7
Multiracial	8.0	7.9	8.0
Hispanic non-White	19.3	22.8	16.1
Missing	1.0	0.0	1.8
Sexual orientation, %			
Straight	81.2	80.2	82.1
Bisexual	12.7	12.9	12.5
Gay or lesbian	6.1	6.9	5.4
Five Facet Mindfulness Questionnaire score, mean (SD)	82.4 (12.6)	81.4 (12.5)	83.4 (12.6)
Smoking-related variables			
Count of cigarettes daily	12.3 (6.1)	12.4 (6.7)	12.3 (5.5)
Fagerström Test for Cigarette Dependence score, mean (SD)	4.7 (2.0)	4.9 (2.1)	4.5 (2.0)
Motivation to quit smoking cigarettes, mean (SD)	6.9 (2.4)	6.7 (2.5)	7.1 (2.3)

a There was no statistically significant group difference for any measured variable shown at baseline.

**Figure 2.**
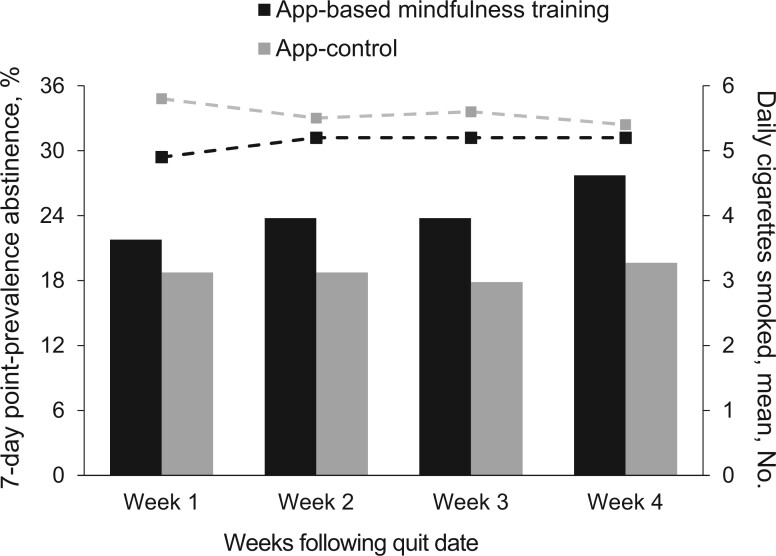
Descriptive statistics for 7-day point-prevalence abstinence and mean number of daily cigarettes, by week and study group. *Point-prevalence abstinence* refers to complete (not even a single puff) 7-day abstinence, quantified by responses made on the timeline follow-back calendar for each calendar day during the quit period. The 7-day point-prevalence abstinence is shown on the left *y*-axis and as bars; the mean number of daily cigarettes is shown on the right *y*-axis and as lines, by week and study group.

After the quit date, 7.0% of the sample used a cessation quit aid, such as gum or patches, even though the study interventions did not actively make this recommendation. Figure 2 provides descriptive statistics for complete 7-day point-prevalence abstinence as well as the daily count of cigarettes smoked, by intervention group, across the 4 weeks of the quit period. Weekly complete 7-day point-prevalence abstinence proportions ranged from 21.8% to 27.7% in the app-based mindfulness training group and 17.9% to 19.6% in controls. The average daily count of cigarettes smoked across 1 week ranged from 4.9 to 5.2 in the app-based mindfulness training group and 5.4 to 5.8 in the control group.

### Study group effect on total abstinence days and complete 7-day point-prevalence abstinence

Table 2 displays the results for the model estimated aggregate group-level contrasts comparing the app-based mindfulness training and control study intervention packages on total abstinence days during the follow-up period (28 in total) and 7-day point-prevalence abstinence for the final week. For total abstinence days, the estimated group effect, adjusting for sex and cigarette dependence (FTCD) at baseline, yielded a significant group effect for the ITT single-dose sample (OR = 2.00, 95% CI = 1.03 to 3.87, *P* = .041; n = 174). The group effect for the ITT randomized sample was of similar magnitude and direction but was at the threshold of statistical significance (OR = 1.90, 95% CI = 0.99 to 3.63, *P* = .052; n = 213). When modeling the final week of follow-up for complete 7-day point-prevalence abstinence, the direction of advantage again favored app-based mindfulness training (OR = 1.65, 95% CI = 0.84 to 3.23, *P* = .148; n = 174; model estimated proportion abstinent by group = 36% for app-based mindfulness training vs 26% for controls), yet the threshold for statistical significance was unmet. The estimated number needed to treat with app-based mindfulness training produced from this adjusted model was 9.5 to produce 1 complete 7-day abstinence case. The sex covariate as an a priori randomization stratum and level of cigarette dependence at baseline as an a priori nicotine addiction magnitude covariate had a null effect on total abstinence days and 7-day point-prevalence abstinence for the final week.

**Table 2.** Estimated group effect on total abstinence days and 7-day point-prevalence abstinence for week 4 of the 28-day quit period

Variable	ITT randomized (n = 213)			ITT single dose or more (n = 174)		
	OR	95% CI	*P*	OR	95% CI	*P*
**Abstinence days during 28-d quit period^a^**						
Study group						
Control	(Referent)	—	—	(Referent)	—	—
App-based mindfulness training	1.90	0.99 to 3.63	.052	2.00	1.03 to 3.87	.041
Sex						
Male	(Referent)	—	—	(Referent)	—	—
Female	1.18	0.62 to 2.27	.614	1.24	0.64 to 2.42	.527
Fagerström Test for Cigarette Dependence^b^	0.91	0.78 to 1.08	.287	0.93	0.79 to 1.10	.411
**Complete 7-d point-prevalence abstinence for week 4 of quit period^a^**						
Study group						
Control	(Referent)	—	—	(Referent)	—	—
App-based mindfulness training	1.65	0.84 to 3.23	.148	1.60	0.81 to 3.18	.177
Sex						
Male	(Referent)	—	—	(Referent)	—	—
Female	1.24	0.63 to 2.45	.536	1.25	0.62 to 2.49	.535
Fagerström Test for Cigarette Dependence^b^	1.03	0.87 to 1.23	.698	1.04	0.87 to 1.24	.645

a Model adjusts for sex as the stratum variable used for randomization and for cigarette dependence. CI = confidence interval; ITT = intent to treat; OR = odds ratio; PPA = complete 7-day point-prevalence abstinence.

b In the Fagerström Test for Cigarette Dependence, higher scores indicate greater dependence.

### Study group effect on the number of cigarettes smoked per day

Table 3 displays the results for the model estimated contrast comparing the app-based mindfulness training and control groups on mean number of cigarettes smoked per day. The estimated group effect, adjusting for sex and cigarette dependence (FTCD) covariate scores at baseline, yielded a significant group effect for the ITT single-dose sample (OR = 0.81, 95% CI = 0.71 to 0.92, *P* = .002; n = 174) and the ITT randomized sample of similar magnitude and significance (OR = 0.87, 95% CI = 0.77 to 0.99, *P* = .030; n = 213). The model estimated mean number of cigarettes per day was 4.95 in the app-based mindfulness training group vs 5.69 in controls. The sex covariate as an a priori randomization stratum had a null effect, whereas the level of cigarette dependence (FTCD) at baseline predicted higher a mean number of cigarettes smoked per day. For the ITT single-dose sample, a 50% reduction in the number of cigarettes smoked was observed in 67.1% of those in the app-based mindfulness training group compared with 57.3% in the control group.

**Table 3.** Estimated group effect on mean number of cigarettes smoked per day during the 28-day quit period

Variable	ITT randomized (n = 213)			ITT single dose^a^ (n = 174)		
	Incidence rate ratio	95% CI	*P*	Incidence rate ratio	95% CI	*P*
Study group						
Control	(Referent)	—	—	(Referent)	—	—
App-based mindfulness training	0.87	0.77 to 0.99	.030	0.81	0.71 to 0.92	.002
Quit days	1.00	1.00 - 1.00	.887	1.00	1.00 - 1.00	.588
Sex^b^						
Male	(Referent)	—	—	(Referent)	—	—
Female	0.93	0.82 to 1.05	.250	0.92	0.81 to 1.05	.202
Fagerström Test for Cigarette Dependence^c^	1.24	1.20 to 1.29	<.001	1.24	1.20 to 1.29	<.001

a For the ITT single-dose sample, a 50% reduction in cigarettes smoked was observed in 67.1% of the Mindfulness Training app group, as opposed to 57.3% in the control group. CI = confidence interval; ITT = intent to treat.

b Model adjusts for sex as the stratum variable used for randomization and for cigarette dependence.

c In the Fagerström Test for Cigarette Dependence, higher scores indicate greater dependence.

### Group effect on time to first cigarette smoked after a quit attempt

In a supplementary descriptive analysis to determine whether smoking abstinence contained a time element that was a function of study group, we produced a Kaplan-Meier curve to assess days from quit date to first cigarette smoked. The overall median abstinence days for the total sample was 24. The median abstinence days to first cigarette smoked after the quit day showed an advantage in the app-based mindfulness training group at 24 days compared with controls at 21 days, yet the group contrast did not reach statistical significance (log-rank *P* = .66).

### Use of intervention sessions and relation to proportion of days abstinent

**Figure 3.**
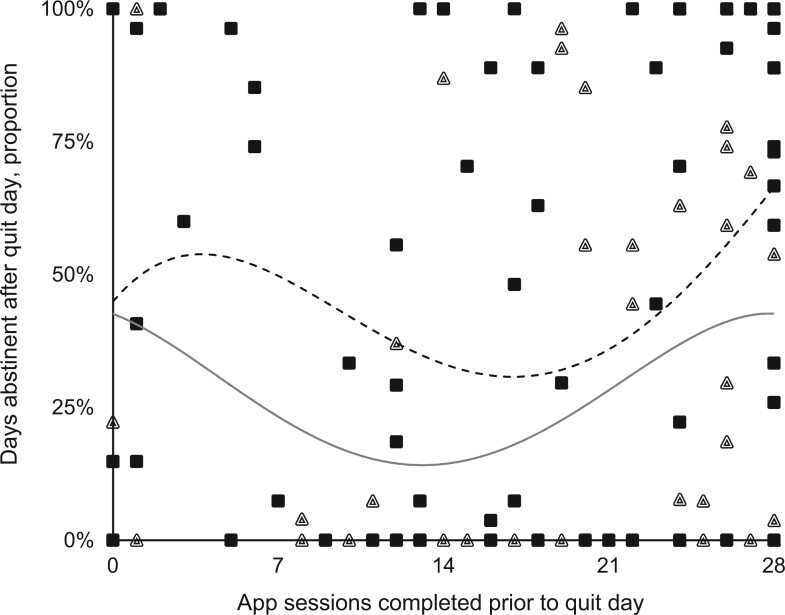
Relation between the number of intervention sessions completed before the quit date and abstinence days during the quit period, by group. App-based mindfulness training = **black box**; control = **triangle**; n = 154 participants having data for both the *x*-axis and *y*-axis variables. The **dashed curved line** is the app-based mindfulness training polynomial trend line; the **solid curved line** is the control polynomial trend line. Among the sample participants who completed more than 20 app sessions, the average proportion of days abstinent in the app-based mindfulness training group was more than double that of controls (56% vs 36%, respectively).

The number of app sessions completed in the app-based mindfulness training group was significantly less than controls (15.6 vs 19.9 app sessions completed, *t* = 3.30, *P* = .001; n = 213). The Headspace interface provided the number of minutes each participant completed only meditation practice in open sessions but omitted the minutes for each session’s didactic preamble of instructions, resulting in a method artifact likely responsible for this use discrepancy by group. For the app-based mindfulness training group, among 86 participants, 26.7% completed 0 to 7 sessions, 19.8% completed 8 to 14 sessions, 17.5% completed 15 to 21 sessions, and 36.0% completed 22 to 28 sessions. Among 96 controls, 8.3% completed 0 to 7 sessions, 15.7% completed 8 to 14 sessions, 22.9% completed 15 to 21 sessions, and 53.1% completed 22 to 28 sessions. Figure 3 plots the descriptive statistics for study app sessions completed in relation to the proportion of days abstinent, by group. The descriptive least squares polynomial fit line suggests an upturn in the proportion of abstinence days after the completion of 20 sessions, with the positive slope higher for the app-based mindfulness training group. We used this data-driven analysis to overlay descriptive means (SDs) by group for participants completing more than 20 sessions vs 20 or fewer sessions. The mean proportion of days abstinent among participants completing more than 20 sessions was 56% in the app-based mindfulness training group and 36% in controls, a difference of 20%.

## Discussion

In this registered randomized controlled study, structured by a 2-week intervention period preceding a voluntary planned smoking quit date, we tested the effect of an app-based mindfulness training intervention compared with an attention control on smoking behavior during a 28-day quit attempt period. The trial results suggest that among the sample participants who opened apps at least once (a single dose), the app-based mindfulness training group showed an advantage on proportion of total days abstinent during a quit attempt period. The model estimated advantage of app-based mindfulness training on 7-day point-prevalence abstinence at the last week of assessment (week 4 of the quit period), however, did not reach statistical significance. In practical terms, our model estimated that it would require about 10 smokers willing to quit in the context of a trial to produce 1 successful complete 7-day (point-prevalence abstinence) abstinence case by week 4 of a quit attempt period. Further, the app-based mindfulness training group showed an advantage with respect to fewer average number of cigarettes smoked per day across the quit period. This pattern of results was initially promising, especially given that the Headspace app used in the app-based mindfulness training group is already fully developed, available to the public, and can be accessed by people in remote settings. Although the evidence yielded from this trial is limited for interpreting complete 7-day point-prevalence abstinence at week 4 of a quit attempt, our results are more promising for smoking reduction, which is considered an important harm-reduction strategy to reduce exposure to the carcinogenic properties of cigarette smoke and for the prevention of tobacco-related diseases (37).

To address a gap in the field, we also used a data-driven approach to assess the number of study app sessions completed in relation to the proportion of days abstinent to attempt to decipher a target number of app sessions that might be productive for future studies using this mindfulness app or similar app types. This tentative finding suggests that among the sample participants who completed more than 20 app sessions, the average proportion of days abstinent in the app-based mindfulness training group was more than double that of controls (56% vs 36%, respectively). If this descriptive finding maintains generality for other samples, it offers users a clear goal of completing 20 app sessions, which equates to about 3.5 hours of commitment across 14 days. This finding is helpful to research and practice domains given that concrete recommendations for dosing mindfulness training are not well established, especially when delivered by app (38).

We presumed that mindfulness meditation is the independent variable to function on smoking behavior as the dependent variable given that our selected attention control mirrors the intervention package on daily time exposure to digital educational content, study protocols, staff contacts, assessments, monitoring, and compensation schedules. All participants also received the self-administered smoking cessation workbook developed by the National Cancer Institute to hold constant the existing minimum standard of support offered to the public at no cost. Thus, there is a basic level of quit smoking education offered to all participants in the trial, including the education offered to all participants during our single motivational interview session at baseline, conducted by our trained research staff. We suspected that adding an app-based mindfulness training intervention assigned daily, twice per day, for 2 weeks offered a preparatory period to practice mindfulness training and gain skills in avoiding a response to fluctuating states, including aversive states associated with not smoking and deprivation of tobacco and/or nicotine. We specifically selected the Headspace app for the mindfulness training group because it focuses on guided meditation content not adapted for smoking cessation to determine whether a generalized mindfulness training app offers a benefit beyond what has previously been found for mindfulness training programs specifically adapted to smoking (20).

Our findings suggest that mindfulness training offers a benefit without being specific to smoking behavior. A future study comparing a generalized mindfulness training app, such as Headspace, with smoking-specific mindfulness training intervention packages appears to be warranted at this time to determine the level of equivalence in their effects on smoking outcomes. Smokers could have the choice to select either the general mindfulness training or mindfulness training focused on smoking, depending on their preference and tolerance for educational material on smoking.

The design characteristics of this randomized controlled trial contribute to the general strength of our study. For example, our remote research protocols for recruitment, interviewing, and surveying can be replicated at relatively low cost in future studies designed to examine longer periods of mindfulness training or mindfulness training combined with pharmacotherapy, such as nicotine replacement therapy. In support of this future aim, previous studies have identified telehealth approaches (eg, delivery of quit-smoking counseling by telephone) combined with nicotine replacement therapy to be a cost-effective strategy for cessation in clinical settings (39). Our interpretations of this trial’s results are limited in a few ways. First, any findings from the current study cannot be generalized to individuals not willing to use apps. This limitation is evident in some older adults who are unfamiliar with smartphone technology and those who lack access to a personal smartphone or are unwilling to use smartphone apps. Second, smoking abstinence was not biologically verified with carbon monoxide testing because of budget constraints. Our previous work indicated that self-report measurement of smoking is reliable (40), so we do not expect major group differences in the veracity of self-report, although it is possible. Third, any detected effect by group in this trial may be the result of extraneous factors that our assessment strategy did not uncover. For example, instructions in the app-based mindfulness training group may have led participants to initiate other behaviors as part of lifestyle modification, such as changes in yoga-based physical activity or in social relationships (eg, seeking out people who do not smoke to spend time with), which are not directly attributable to our presumed independent variable (ie, Headspace app–guided mindfulness meditation).

Fourth, given the nature of the behavior intervention, which included practice and skill development, participants became aware of their study group content immediately following trial enrollment, which is a ubiquitous artifact of behavior intervention research. Unblinded study group content in this way can differentially reinforce participant adherence to select or preferred study interventions and protocols. Fifth, participants received compensation after the intervention period for the number of app session they completed, which may have increased their motivation to complete sessions. The total possible incentive amount was small ($2.50 per session), however, and compensation was not paid until the end of the intervention period. Compensation schedules were the same in both study groups. Sixth, the post-quit date follow-up period did not include assessment of additional app use or sustained abstinence at 3 and 6 months. This limited follow-up period masked the potential to detect lagged effects on smoking, effects that increase or decrease over time, and null effects that persist over time. Finally, our ITT analysis, which modeled the 7-day point-prevalence abstinence outcome, could be underpowered, as the actual missing data at the final 7-day point-prevalence abstinence period was 19% (162/200), which was higher than the expected 10% (180/200) we used in our pretrial power analysis.

In summary, we find that low-demand, daily, app-guided mindfulness training with Headspace for the 2 weeks leading up to a planned quit date shows an empirical advantage over an attention control that includes basic education for smoking cessation, resulting in fewer days smoked and fewer cigarettes smoked in a sample of regionally diverse adults willing to make a quit attempt. These findings have implications for the use of commercially available mindfulness training apps that can be widely disseminated by health workers to potentially reducing exposure to the carcinogenic properties of inhaled cigarette smoke.

## Supplementary Material

pkad095_Supplementary_Data

## Data Availability

Data are provided in an online supplement accompanying this article, as published by the Journal.
